# Supplementary material for: Measuring transcription factor binding and gene expression using barcoded self-reporting transposon calling cards and transcriptomes
Source: NAR Genom Bioinform. 2022 Aug 31;4(3):lqac061. doi: 10.1093/nargab/lqac061 (PMC9428926; doi:10.1093/nargab/lqac061)
Supplement: lqac061_Supplemental_File [file lqac061_supplemental_file.docx]

**Supplementary Material for Lalli *et al.* Barcoded Calling Cards**


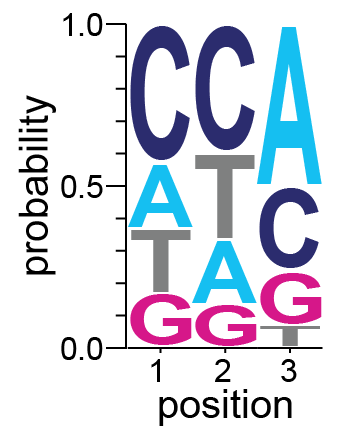


**Supplementary Figure 1:** Sequence logo of the top 30 most abundantly inserted 3-nt barcoded SRTs reveals modest sequence preference for integration efficiency.


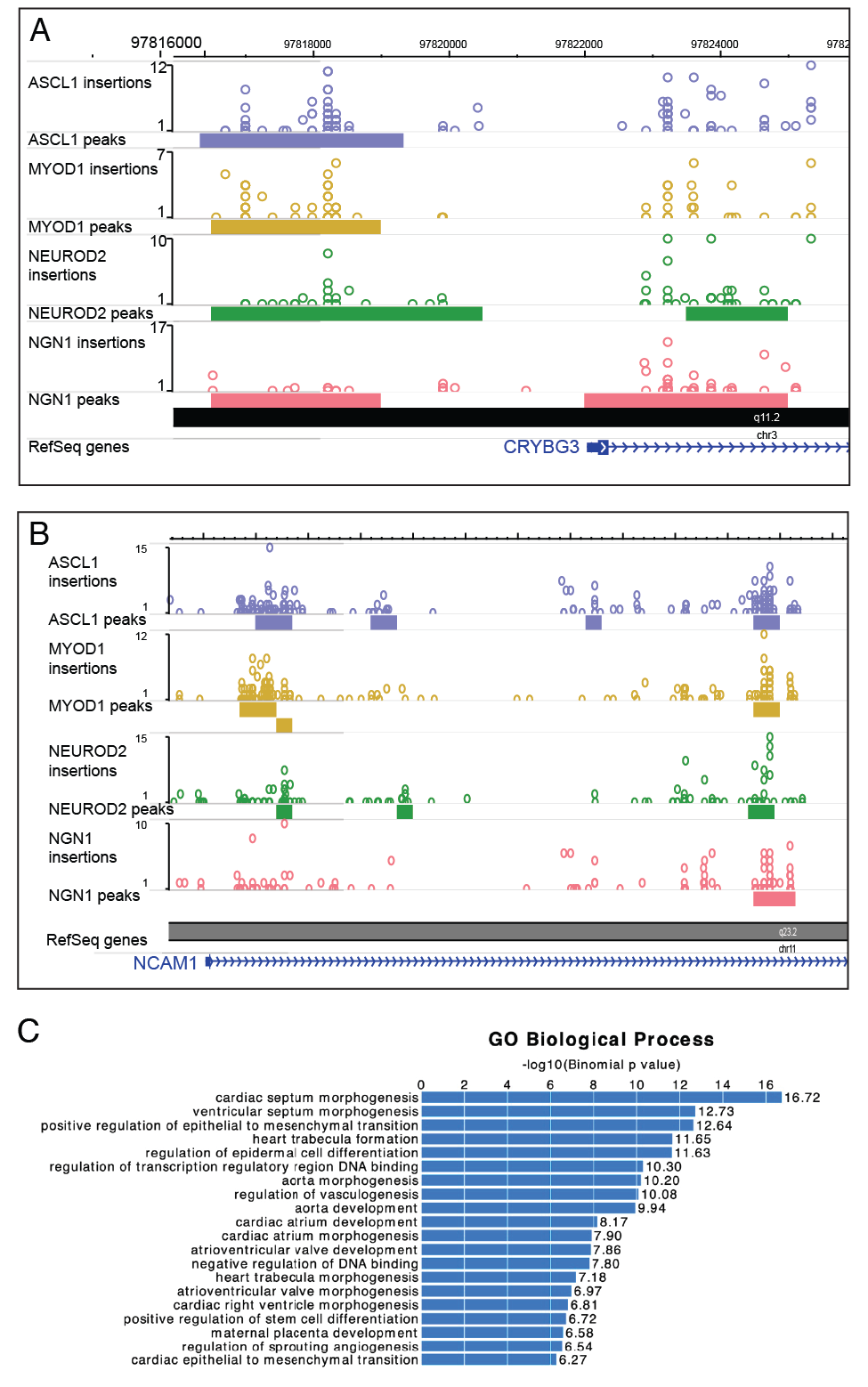


**Supplementary Figure 2:** Calling card insertions and peaks for four transcription factors at shared and distinct genes. Browser views of genomic insertions and called peaks highlight binding sites shared across all factors at A) *CRYBG3* and B) *NCAM1*. C) GREAT analysis of MYOD1 hops identifies pathways enriched in heart development.


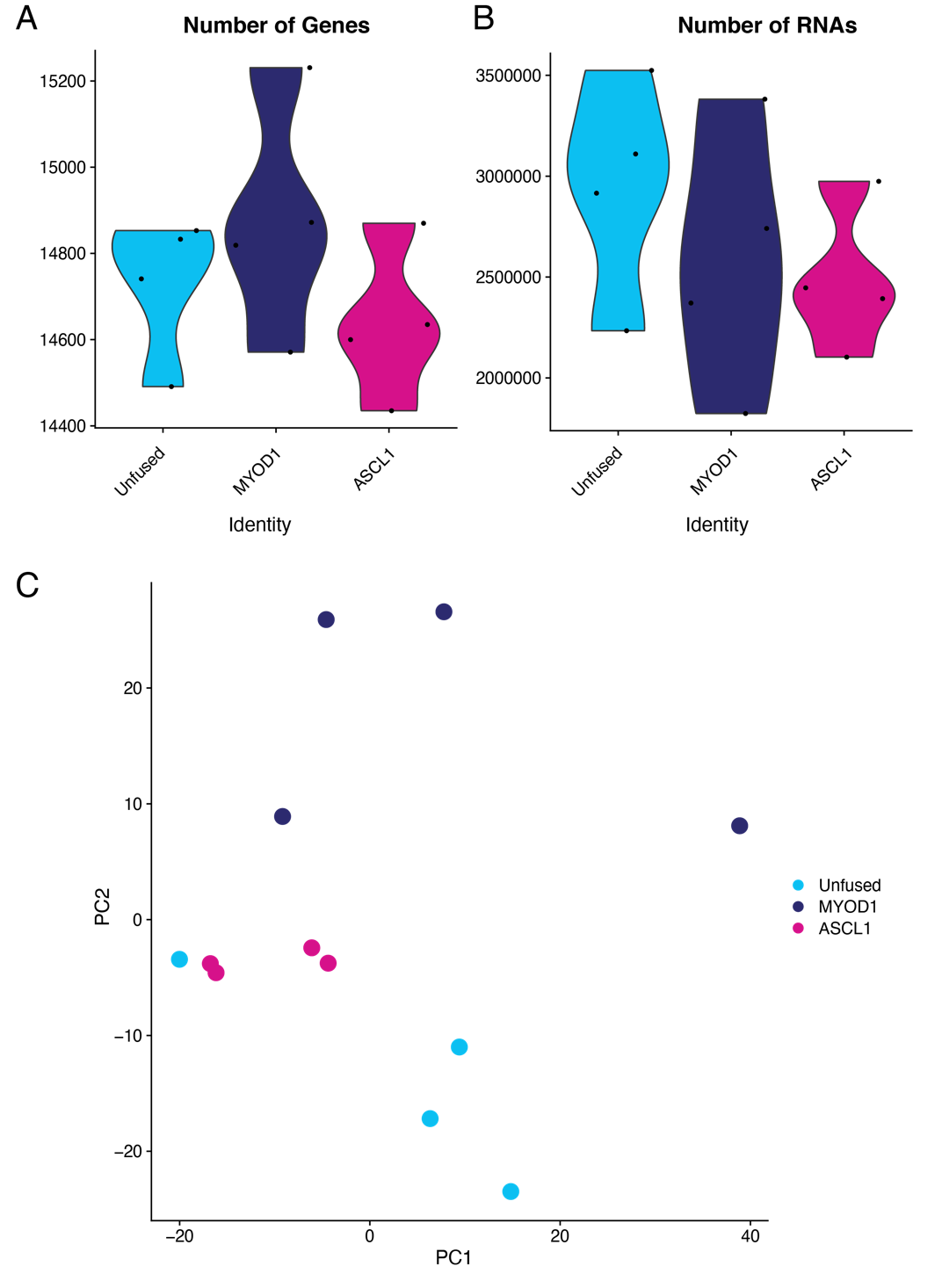


**Supplementary Figure 3:** Quality control of Bulk Barcoded RNA sequencing samples. A) Number of genes detected in each sample. B) Total number of RNAs collapsed by unique molecular identifiers. All 12 samples, which were prepared and sequenced as a single pool, were present and contained approximately the same number of genes and RNAs detected. C) Principal component analysis for dimensionality reduction and visualization of samples shows clustering by experimental subgroup.

| Sequence | Number of Events |
| --- | --- |
| TT*CTAGGG | 9256 |
| TT**C**CTAGGG | *6430* |
| TT**C**CTAGG_ | 5476 |
| TT*CTA**C**GG | 3878 |
| TT*CT**C**GGG | 2870 |
| TT**G**CTAGGG | *2748* |
| TT*CT**G**GGG | 2570 |
| TT*CTA**A**GG | 2202 |
| TT*CT**T**GGG | 2019 |
| TT*CTA**T**GG | 1887 |
| TT**G**CTAGG_ | *1625* |

**Supplementary Table 1:** piggyBac TR does not accommodate a 1-nt insertion at position marked by asterisk (*). Wild-type sequence (red). Sequences with 1-nt insertion (italics) also had 1-nt deletion in 2/3 cases. Mutations to wild-type sequences are **bolded.** Underlines mark single nt deletions.

| Barcode Number | Sequence |
| --- | --- |
| 1 | CAAC |
| 2 | CTGA |
| 3 | GCAT |
| 4 | GTAC |
| 5 | CACA |
| 6 | TGAC |
| 7 | GTCA |
| 8 | CGAT |
| 9 | CTCT |
| 10 | TCGA |
| 11 | GAAG |
| 12 | CATG |
| 13 | GTTG |
| 14 | CTTC |
| 15 | GCTA |
| 16 | GAGA |
| 17 | GTGT |
| 18 | CGTA |
| 19 | TGGT |
| 20 | GGAA |
| 21 | ACAC |
| 22 | TCAG |
| 23 | TTGG |
| 24 | CAGT |
| Wild-type | CTAG |

**Supplementary Table 2:** The top 24 integration-competent error-detecting barcodes were cloned into two self-reporting transposon vectors: an adeno-associated viral (AAV) vector carrying a tdTomato reporter SRT and a non-AAV SRT vector encoding the puromycin resistance gene. The wild-type sequence of the 13-bp terminal repeat is: TATCTTT**CTAG**GG. Underlined bases were mutagenized to the listed barcode sequences.

| Replicate | Transcription Factor | Self-Reporting Transposon | Hops | Total Hops | Called Peaks |
| --- | --- | --- | --- | --- | --- |
| 1 | Unfused hyperPBase | WT PB-SRT-Puro | 191776 |  |  |
| 2 | Unfused hyperPBase | WT PB-SRT-Puro | 161056 |  |  |
| 3 | Unfused hyperPBase | WT PB-SRT-Puro | 222416 | 643632 | N/A |
| 1 | NEUROD2 | WT PB-SRT-Puro | 201986 |  |  |
| 2 | NEUROD2 | WT PB-SRT-Puro | 192798 |  |  |
| 3 | NEUROD2 | WT PB-SRT-Puro | 213119 |  |  |
| 4 | NEUROD2 | WT PB-SRT-Puro | 245030 | 852501 | 4594 |
| 1 | ASCL1 | Barcoded PB-SRT-tdTomato | 207857 |  |  |
| 2 | ASCL1 | Barcoded PB-SRT-tdTomato | 207771 |  |  |
| 3 | ASCL1 | Barcoded PB-SRT-tdTomato | 280185 |  |  |
| 4 | ASCL1 | Barcoded PB-SRT-tdTomato | 316377 | 1011703 | 4027 |
| 1 | MYOD1 | Barcoded PB-SRT-tdTomato | 305021 |  |  |
| 2 | MYOD1 | Barcoded PB-SRT-tdTomato | 302416 |  |  |
| 3 | MYOD1 | Barcoded PB-SRT-tdTomato | 246855 | 853965 | 6178 |
| 1 | NGN1 | Barcoded PB-SRT-puro pool | 189488 |  |  |
| 2 | NGN1 | Barcoded PB-SRT-puro pool | 216185 |  |  |
| 3 | NGN1 | Barcoded PB-SRT-puro pool | 254215 |  |  |
| 4 | NGN1 | Barcoded PB-SRT-puro pool | 249453 | 904991 | 3593 |

**Supplementary Table 3:** Description of Calling Card Experiments. All experiments had ≥ 3 replicate transfections. Self-reporting transposon reporter gene is indicated. Number of genomic insertions (hops). N/A, not applicable. Genomic insertions of unfused hyper-piggyBac are used as the background to call peaks. per replicate are indicated. Cumulative number of hops for each transcription factor, and number of called peaks are shown.

| Transcription Factor | Self-Reporting Transposon | Reads | Hops | Peaks* |
| --- | --- | --- | --- | --- |
| ASCL1 | Barcoded PB-SRT-puro pool | 3865901 | 532995 | 4571 |
| MYOD1 | Barcoded PB-SRT-puro pool | 3083682 | 587277 | 7537 |
| Unfused | Barcoded PB-SRT-puro pool | 3513592 | 362896 | N/A |

**Supplementary Table 4:** Description of Calling Card and Transcriptome Experiments. Self-reporting transposon reporter, number of total sequencing reads, number of uniquely mapped reads, and number of genomic insertions (hops) are indicated. N/A, not applicable. *Genomic insertions of unfused hyper-piggyBac from the previous experiments (Supplementary Table 3) were used as the background to call peaks.

| **Primer Name** | **Primer Sequence** |
| --- | --- |
| pSeq1-BC1-UMI-dtVN | CTACACGACGCTCTTCCGATCTCTGATAGCATGGTCATNNNNNVVVVVTTTTTTTTTTTTTTTTTTTTTTTTTTTTTTVN |
| pSeq1-BC2-UMI-dtVN | CTACACGACGCTCTTCCGATCTCACAGTAGTTAGGGTGNNNNNVVVVVTTTTTTTTTTTTTTTTTTTTTTTTTTTTTTVN |
| pSeq1-BC3-UMI-dtVN | CTACACGACGCTCTTCCGATCTGTAACTGCATGGTCTANNNNNVVVVVTTTTTTTTTTTTTTTTTTTTTTTTTTTTTTVN |
| pSeq1-BC4-UMI-dtVN | CTACACGACGCTCTTCCGATCTACTGAACCAGTGGGATNNNNNVVVVVTTTTTTTTTTTTTTTTTTTTTTTTTTTTTTVN |
| pSeq1-BC5-UMI-dtVN | CTACACGACGCTCTTCCGATCTAACACGTTCAGTTCGANNNNNVVVVVTTTTTTTTTTTTTTTTTTTTTTTTTTTTTTVN |
| pSeq1-BC6-UMI-dtVN | CTACACGACGCTCTTCCGATCTTATCAGGGTTTAGCTGNNNNNVVVVVTTTTTTTTTTTTTTTTTTTTTTTTTTTTTTVN |
| pSeq1-BC7-UMI-dtVN | CTACACGACGCTCTTCCGATCTACTTTCATCGTAGGAGNNNNNVVVVVTTTTTTTTTTTTTTTTTTTTTTTTTTTTTTVN |
| pSeq1-BC8-UMI-dtVN | CTACACGACGCTCTTCCGATCTAACGTTGGTAGCGTCCNNNNNVVVVVTTTTTTTTTTTTTTTTTTTTTTTTTTTTTTVN |
| pSeq1-BC9-UMI-dtVN | CTACACGACGCTCTTCCGATCTCTCATTACAGACGCCTNNNNNVVVVVTTTTTTTTTTTTTTTTTTTTTTTTTTTTTTVN |
| pSeq1-BC10-UMI-dtVN | CTACACGACGCTCTTCCGATCTTGACTAGCAGGGTTAGNNNNNVVVVVTTTTTTTTTTTTTTTTTTTTTTTTTTTTTTVN |
| pSeq1-BC11-UMI-dtVN | CTACACGACGCTCTTCCGATCTGCGGGTTAGTAATCCCNNNNNVVVVVTTTTTTTTTTTTTTTTTTTTTTTTTTTTTTVN |
| pSeq1-BC12-UMI-dtVN | CTACACGACGCTCTTCCGATCTTCTCATAGTTGTGGAGNNNNNVVVVVTTTTTTTTTTTTTTTTTTTTTTTTTTTTTTVN |

**Supplementary Table 5:** Barcoded oligoDT-VN oligos for BRB-seq. We designed barcoded oligoDT-VN oligos to mimic 10x Genomics v2 chemistry: partial seq1, 16 bp cell barcode extracted randomly from the 10x Genomics safelist (737K-august-2016.txt), and a 10 bp UMI (5N + 5V). “Cell barcodes” that serve as sample identifiers are underlined.

| Primer Name | Primer Sequence |
| --- | --- |
| P5_BC_SRT_STAGGER1 | AATGATACGGCGACCACCGAGATCTACAC XXXXXX ACACTCTTTCCCTACACGACGCTCTTCCGATCT T GCGTCAATTTTACGCAGACTATCTTT |
| P5_BC_SRT_STAGGER3 | AATGATACGGCGACCACCGAGATCTACAC XXXXXX ACACTCTTTCCCTACACGACGCTCTTCCGATCT GCA GCGTCAATTTTACGCAGACTATCTTT |
| P5_BC_SRT_STAGGER5 | AATGATACGGCGACCACCGAGATCTACAC XXXXXX ACACTCTTTCCCTACACGACGCTCTTCCGATCT CTTAG GCGTCAATTTTACGCAGACTATCTTT |
| P5_BC_SRT_STAGGER7 | AATGATACGGCGACCACCGAGATCTACAC XXXXXX ACACTCTTTCCCTACACGACGCTCTTCCGATCT TACTTAT GCGTCAATTTTACGCAGACTATCTTT |
| P5_BC_SRT_STAGGER9 | AATGATACGGCGACCACCGAGATCTACAC XXXXXX ACACTCTTTCCCTACACGACGCTCTTCCGATCT AGGCATGAT GCGTCAATTTTACGCAGACTATCTTT |
| P5_BC_SRT_STAGGER11 | AATGATACGGCGACCACCGAGATCTACAC XXXXXX ACACTCTTTCCCTACACGACGCTCTTCCGATCT GATAGCGTGCG GCGTCAATTTTACGCAGACTATCTTT |

**Supplementary Table 6:** Barcoded SRT library primers with stagger regions to introduce sequence diversity to Read 1. We recommend sequencing at least 4 SRT libraries constructed with different stagger lengths on the same flow cell and using 20% PhiX DNA spike-in. Stagger region is underlined. XXXXXX denotes i5 index.
